# Cross-Cultural Validation of Mood Profile Clusters in a Sport and Exercise Context

**DOI:** 10.3389/fpsyg.2018.01949

**Published:** 2018-10-09

**Authors:** Alessandro Quartiroli, Renée L. Parsons-Smith, Gerard J. Fogarty, Garry Kuan, Peter C. Terry

**Affiliations:** ^1^Department of Psychology, University of Wisconsin-La Crosse, La Crosse, WI, United States; ^2^Division of Research and Innovation, University of Southern Queensland, Toowoomba, QLD, Australia; ^3^School of Social Sciences, University of the Sunshine Coast, Sippy Downs, QLD, Australia; ^4^School of Health Sciences, Universiti Sains Malaysia, Kelantan, Malaysia; ^5^School of Medical Sciences, Universiti Sains Malaysia, Kelantan, Malaysia

**Keywords:** affect, emotions, ITAMS, BRUMS, cluster analysis, mood profiling, online assessment

## Abstract

Mood profiling has a long history in the field of sport and exercise. Several novel mood profile clusters were identified and described in the literature recently (Parsons-Smith et al., 2017). In the present study, we investigated whether the same clusters were evident in an Italian-language, sport and exercise context. The Italian Mood Scale (ITAMS; Quartiroli et al., 2017) was administered to 950 Italian-speaking sport participants (659 females, 284 males, 7 unspecified; age range = 16–63 year, *M* = 25.03, *SD* = 7.62) and seeded *k*-means clustering methodology applied to the responses. Six distinct mood profiles were identified, termed the iceberg, inverse iceberg, inverse Everest, shark fin, surface, and submerged profiles, which closely resembled those reported among English-speaking participants (Parsons-Smith et al., 2017). Significant differences were found in the distribution of specific mood profiles across gender and age groups. Findings supported the cross-cultural generalizability of the six mood profiles and offer new research avenues into their antecedents, correlates and behavioral consequences in Italian-language contexts.

## Introduction

Mood has been defined as “a set of feelings, ephemeral in nature, varying in intensity and duration, and usually involving more than one emotion” (Lane and Terry, 2000, p. 17). Relationships between mood and performance have been studied extensively across several performance domains, including various sporting contexts (Beedie et al., 2000), school examinations (Lane et al., 2005), and cognitive tasks (Nadler et al., 2010; Lane et al., 2017). Other research has focused on more theoretical goals, such as distinguishing moods from emotions (Watson and Clark, 1994; Ekman, 1999; Beedie et al., 2005), establishing the multidimensional structure of moods (Lane and Terry, 2000; Crawford and Henry, 2004), developing measures of mood (McNair et al., 1971, 1992; Watson et al., 1988; Terry et al., 1999), and testing the validity of mood measures across different cultural settings (Terry et al., 2003b, 2012; Rohlfs et al., 2005 Zhang et al., 2014).

Mood profiling is a method of identifying and capturing commonly occurring mood combinations (Parsons-Smith et al., 2017). Using this methodology, individuals can be assigned to groups, with group membership, rather than individual scores on mood dimensions, being used as a predictor of behavioral outcomes, including performance. Morgan (1980) was among the first to use mood profiling in sport. He based his work on the six mood dimensions identified by McNair et al. (1971); namely, tension, depression, anger, vigor, fatigue, and confusion. Morgan (1980) postulated a mental health model that predicted an inverse relationship between psychopathology and sport performance. Moreover, he proposed that successful performers tended to report a mood profile that differed from population norms, which he termed the *iceberg* profile, characterized by an above average score for vigor and below average scores for tension, depression, anger, fatigue, and confusion (Morgan, 1980).

The iceberg profile has since been investigated in many studies (see Rowley et al., 1995; Beedie et al., 2000) and has also stimulated a search for other profiles. An early addition was the *Everest* profile, which is characterized by significantly higher levels of vigor and lower levels of tension, depression, anger, fatigue, and confusion than the iceberg profile, and is proposed to be associated with superior athletic performance (Terry, 1995). Another profile, the *inverse iceberg*, is characterized by below average vigor, and above average tension, depression, anger, fatigue, and confusion scores, and has been shown to be associated with underperformance (Budgett, 1998), incidence of injury (Galambos et al., 2005), and risk of psychopathology (van Wijk et al., 2013).

More recently, additional profiles have been identified. Parsons-Smith et al. (2017) interrogated 2,364 responses to an online mood profiling system, known as *In The Mood* ^1^ (Terry and Lim, 2011). They identified six profiles, which included the iceberg and inverse iceberg profiles, and also described four novel profiles, termed the inverse Everest, surface, shark fin, and submerged profiles, which were cross-validated on two large independent samples. The *inverse Everest* profile was characterized by low vigor, high tension and fatigue, and very high depression, anger, and confusion scores. The *surface* profile was characterized by near average scores on all six mood dimensions. The *shark fin* profile was characterized by below average scores for tension, depression, anger, vigor, and confusion, combined with a high level of fatigue, while the *submerged* profile was characterized by below average scores on all six dimensions of mood.

Mood profiling is an important technique in applied settings, such as sport, because it identifies from among a very large set of possible combinations of scores only those occurring on a reasonably frequent basis. Associations between particular profiles and various outcome variables can then be explored. If some profiles, such as the Everest and iceberg, are associated with superior performance, coaches and athletes can attempt to generate the desired pattern of mood dimensions in competitive situations. Alternatively, profiles associated with poor performance, which logically would include the shark fin and submerged profiles, and those indicating risk of psychopathology, such as the inverse Everest and inverse iceberg profiles, may signal the need for mood regulation strategies to be implemented (Larsen, 2009) or referral to a mental health professional.

To date, research on the expanded set of profiles identified by Parsons-Smith et al. (2017) has been restricted to English-speaking populations. For these profiles to be generalizable, they would have to demonstrate stability and replicability across different cultural settings. Whether the same mood profiles are identifiable in non-English-speaking countries is a question yet to be answered. The aim of the current study was to explore mood profile clusters among a sample of Italian-speaking sport participants and to compare the emergent profiles to those found by Parsons-Smith et al. (2017). Confirmation of the novel mood profile clusters in a different cultural context will provide further support for the validity of the profiles, extend the range of psychological assessment techniques available to psychologists in Italian-speaking communities, and stimulate other cross-cultural research into mood states.

## Materials and Methods

### Participants

Two archival datasets were used in the present study. Dataset 1, collected during the development and validation of the Italian Mood Scale (Quartiroli et al., 2017), was derived from 950 Italian-speaking residents of Italy, who regularly engaged in recreational and/or competitive physical activities (659 females, 284 males, 7 unspecified; age range = 16–63 year, *M* = 25.03, *SD* = 7.62). Several types of physical activity were represented, with football, track and field, swimming, wrestling, and volleyball having the highest representation.

Dataset 2 was derived from 2,364 English-speaking adult participants (1,219 males, 1,145 females; age range = 18–65+ year) who were socio-demographically heterogeneous, encompassing a range of education levels and ethnicities. Dataset 2 was originally collected by Lim (2011, unpublished) and was one of three datasets interrogated previously to identify mood profile clusters among the general population of English speakers (Parsons-Smith et al., 2017).

### Measures

#### Italian Mood Scale (ITAMS)

The ITAMS is an Italian-language version of the BRUMS (Terry et al., 1999, 2003a; Quartiroli et al., 2017) that includes four mood descriptors to assess each of six dimensions of mood: anger, confusion, depression, fatigue, tension, and vigor. Respondents indicated their feelings on a 5-point response scale ranging from 0 (*not at all/per nulla*) to 4 (*extremely/moltissimo*) rating “*How do you feel right now/come lei si sente in questo preciso momento*” for each mood descriptor. Total subscale scores ranging from 0 to 16 were computed for each of the six subscales, where higher scores represent stronger endorsement of each mood dimension. The validation process of the ITAMS confirmed the fit of the 6-factor measurement model (CFI = 0.98, TLI = 0.97, RMSEA = 0.05) and supported its concurrent validity against Italian-language versions of the Positive and Negative Affect Schedule (PANAS; Terracciano et al., 2003) and the Depression Anxiety Stress Scales-21 (DASS-21; Bottesi et al., 2015). The internal consistency of the subscales was supported, with Cronbach alpha coefficients ranging from 0.77 to 0.86 for the six subscales (Quartiroli et al., 2017).

#### Brunel Mood Scale (BRUMS)

The BRUMS was used to assess mood responses among English-speaking participants, using a response timeframe of *“How do you feel right now?”* (Terry et al., 2003a). The 24 mood descriptors are also rated on a 5-point Likert scale from 0 (*not at all*) to 4 (*extremely*), with subscale scores for anger, confusion, depression, fatigue, tension, and vigor ranging from 0 to 16. The BRUMS measurement model was validated using multi-sample confirmatory factor analysis across four large samples (Terry et al., 2003a) and the internal consistency of the subscales was supported, with Cronbach alpha coefficients ranging from 0.74 to 0.90 for the six subscales (Terry et al., 1999).

### Procedure

This study was carried out in accordance with the recommendations of the American Psychological Association and the Australian Code for the Responsible Conduct of Research. The protocol was approved by both the Institutional Review Board for the Protection of Human Subjects at the University of Wisconsin – La Crosse and the Human Research Ethics Committee at the University of Southern Queensland (approval number: H11REA023). All participants gave written informed consent in accordance with the Declaration of Helsinki.

Dataset 1 was collected using an anonymous online survey hosted on a Qualtrics platform (Qualtrics, Provo, UT, United States) distributed via social network groups to students at four Italian higher education institutions, with reminder emails posted at 3-week intervals (Dillman et al., 2014). Dataset 2 was collected via the *In The Mood* website (see text footnote^1^; Terry and Lim, 2011) using snowball sampling techniques on social networks (Lim, 2011, unpublished).

### Data Analysis

Parsons-Smith et al. (2017) previously interrogated Dataset 2 (*N* = 2,364) using hierarchical cluster analysis, followed by a *k*-means clustering methodology, identifying six distinct mood profile clusters: iceberg profile, inverse Everest profile, inverse iceberg profile, shark fin profile, submerged profile, and surface profile. In the present study, cluster analysis procedures were applied to Dataset 1 (*N* = 950) to investigate whether the same six mood profiles identified by Parsons-Smith et al. (2017) could be recovered in an Italian-speaking sample. Cluster analysis describes a collection of exploratory data techniques. Hierarchical approaches better delineate natural groups undefined *a priori*, while *k*-means clustering is superior in determining exclusive cluster membership. Where preliminary information about the cluster metrics is known, the *k*-means procedure is generally preferred (Wagstaff et al., 2001). We used knowledge-based clustering, otherwise known as seeded *k*-means clustering, to optimize the *k*-means algorithm and boost clustering performance (Bair, 2013). We then compared the recovered cluster structures to those identified by Parsons-Smith et al. (2017) to assess external validity (Jain et al., 1999; Jain, 2010). Discriminant function analysis (DFA) was used to provide further evidence of the robustness of the cluster structures. All analyses were conducted using the Statistical Package for the Social Sciences, version 25.

## Results

### Data Screening

A total of 21 cases with missing values for the ITAMS subscales were excluded from the analysis. As found in previous samples (Parsons-Smith et al., 2017), significant univariate non-normality was evident in some subscales (e.g., depression) consistent with typical mood subscale distributions. Negative mood scores tend toward higher numbers at the lower end, and lower numbers at the upper end (Terry et al., 1999). The frequency distributions for skewness and kurtosis were inspected and we concluded that deviations from normal distribution were unlikely to make a substantive difference to the analyses. No trimming of the dataset occurred. A total of 24 multivariate outliers were identified according to a Mahalanobis distance statistic at *p* < 0.001, although a case-by-case visual inspection suggested that response patterns were plausible. Given this, all multivariate outliers were retained, and the final sample in Dataset 1 was *N* = 929.

### Cluster Analysis

**FIGURE 1:**
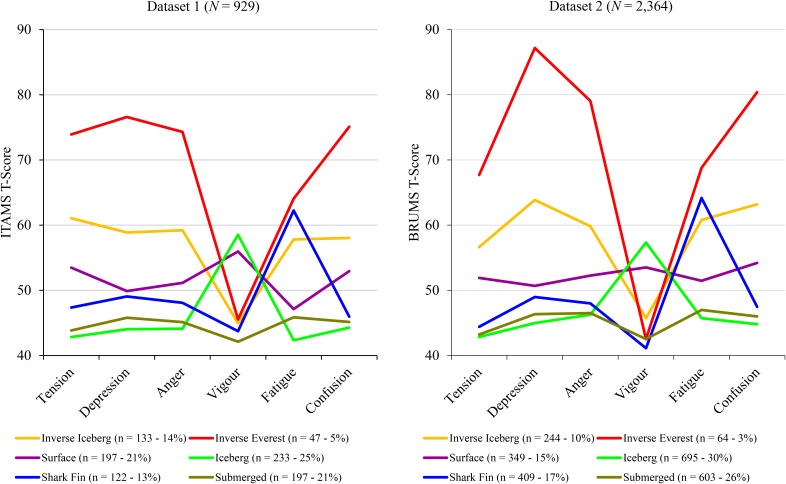
Graphical representation of the 6-cluster solution in two samples.

A seeded *k*-means cluster analysis with a prescribed 6-cluster solution was conducted. Raw score cluster metrics from Dataset 2 provided the initial cluster centroids (**Table 1**). The six clusters identified in Dataset 2 were also recovered in Dataset 1: namely, the iceberg profile, inverse Everest profile, inverse iceberg profile, shark fin profile, submerged profile, and surface profile. **Tables 2**, **3** include descriptive statistics for the 6-cluster solutions for Dataset 1 and Dataset 2, respectively. A graphical representation of the six mood profile clusters for each dataset is shown in **Figure 1**.

**Table 1:** Cluster centroids from Dataset 2 (*N* = 2,364) applied to Dataset 1 (*N* = 929).

	Cluster source					
Mood dimension	1	2	3	4	5	6
Tension	1.15	10.42	6.34	1.75	1.29	4.58
Depression	0.25	11.19	5.11	1.26	0.59	1.69
Anger	0.41	10.23	4.52	0.95	0.48	2.26
Vigor	10.62	4.69	5.98	4.14	4.72	9.10
Fatigue	2.39	11.83	8.59	9.97	2.91	4.76
Confusion	0.54	10.75	5.84	1.32	0.90	3.27

*1, iceberg profile; 2, inverse Everest profile; 3, inverse iceberg profile; 4, shark fin profile; 5, submerged profile; 6, surface profile. Centroids are raw scores.*

**Table 2:** Descriptive statistics of the 6-cluster solution in Dataset 1 (*N* = 929).

	Iceberg profile (*n* = 233)			Inverse Everest profile (*n* = 47)			Inverse iceberg profile (*n* = 133)		
Mood dimension	*M*	*SD*	95% CI	*M*	*SD*	95% CI	*M*	*SD*	95% CI
Tension	42.83	3.86	[42.33, 43.33]	73.91	6.59	[71.98, 75.85]	61.07	6.87	[59.89, 62.25]
Depression	44.02	3.21	[43.61, 44.44]	76.60	11.54	[73.21, 79.98]	58.87	9.02	[57.32, 60.42]
Anger	44.11	3.70	[43.63, 44.59]	74.30	10.97	[71.08, 77.53]	59.21	9.64	[57.56, 60.87]
Vigor	58.53	5.79	[57.78, 59.27]	45.54	10.35	[42.50, 48.58]	44.96	9.04	[43.41, 46.51]
Fatigue	42.34	4.95	[41.70, 42.98]	64.06	10.67	[60.93, 67.20]	57.80	8.69	[56.31, 59.29]
Confusion	44.28	3.81	[43.79, 44.77]	75.09	9.80	[72.21, 77.97]	58.04	9.53	[56.40, 59.67]

	Shark fin profile (*n* = 122)			Submerged profile (*n* = 197)			Surface profile (*n* = 197)		
Mood dimension	*M*	*SD*	95% CI	*M*	*SD*	95% CI	*M*	*SD*	95% CI
Tension	47.36	6.15	[46.25, 48.46]	43.84	4.58	[43.19, 44.48]	53.48	6.03	[52.63, 54.33]
Depression	49.06	6.38	[47.91, 50.20]	45.79	5.10	[45.07, 46.50]	49.88	6.72	[48.94, 50.83]
Anger	48.08	6.84	[46.86, 49.31]	45.13	5.14	[44.40, 45.85]	51.14	6.84	[50.18, 52.10]
Vigor	43.73	7.44	[42.39, 45.06]	42.12	6.31	[41.24, 43.01]	55.96	7.21	[54.94, 56.97]
Fatigue	62.24	6.48	[61.08, 63.40]	45.86	5.33	[45.11, 46.61]	47.12	6.26	[46.24, 48.00]
Confusion	45.97	5.15	[45.05, 46.89]	45.14	4.47	[44.51, 45.77]	52.94	8.03	[51.81, 54.06]

**Table 3:** Descriptive statistics of the 6-cluster solution in Dataset 2 (*N* = 2,364).

	Iceberg profile (*n* = 695)			Inverse Everest profile (*n* = 64)			Inverse iceberg profile (*n* = 244)		
Mood dimension	*M*	*SD*	95% CI	*M*	*SD*	95% CI	*M*	*SD*	95% CI
Tension	42.84	3.59	[42.58, 43.11]	67.70	8.64	[65.54, 69.86]	56.65	7.64	[55.69, 57.61]
Depression	44.98	2.58	[44.79, 45.17]	87.17	11.95	[84.19, 90.16]	63.86	9.95	[62.61, 65.11]
Anger	46.26	2.69	[46.06, 46.47]	79.05	10.81	[76.35, 81.75]	59.82	9.20	[58.66, 60.98]
Vigor	57.33	5.32	[56.93, 57.73]	42.50	10.64	[39.84, 45.16]	45.73	7.54	[44.77, 46.68]
Fatigue	45.72	4.69	[45.37, 46.07]	68.80	7.23	[67.02, 70.58]	60.80	8.38	[59.74, 61.85]
Confusion	44.80	3.38	[44.55, 45.05]	80.39	11.22	[77.59, 83.19]	63.20	8.23	[62.16, 64.24]

	Shark fin profile (*n* = 409)			Submerged profile (*n* = 603)			Surface profile (*n* = 349)		
Mood dimension	*M*	*SD*	95% CI	*M*	*SD*	95% CI	*M*	*SD*	95% CI
Tension	44.42	5.23	[43.91, 44.92]	43.23	4.18	[42.89, 43.56]	51.90	6.10	[51.26, 52.54]
Depression	48.97	6.67	[48.32, 49.62]	46.34	4.75	[45.96, 46.72]	50.68	7.14	[49.93, 51.43]
Anger	48.00	4.58	[47.55, 48.45]	46.50	3.14	[46.25, 46.75]	52.26	7.02	[51.52, 53.00]
Vigor	41.12	6.58	[40.48, 41.76]	42.52	4.67	[42.15, 42.89]	53.51	6.34	[52.85, 54.18]
Fatigue	64.16	6.22	[63.55, 64.76]	46.99	4.51	[46.63, 47.35]	51.46	5.85	[50.84, 52.07]
Confusion	47.47	5.59	[46.93, 48.02]	45.99	4.65	[45.61, 46.36]	54.20	7.16	[53.44, 54.95]

### Discriminant Function Analysis

A *post hoc* simultaneous multiple DFA was used to assess how well the clusters were classified and to further support interpretation and understanding of the cluster solution. DFA predicts membership in naturally occurring groups and is not affected by unequal sample sizes (Tabachnick and Fidell, 2013). DFA is a two-step statistical procedure which involves significance testing of discriminant functions, followed by a computational process of classification. The ratio of cases to independent variables was 155 to 1, which satisfied the preferred requirement of ≥20 to 1. The number of cases in the smallest group was 47, which exceeded the minimum number of cases (i.e., ≥20) per group.

**FIGURE 2:**
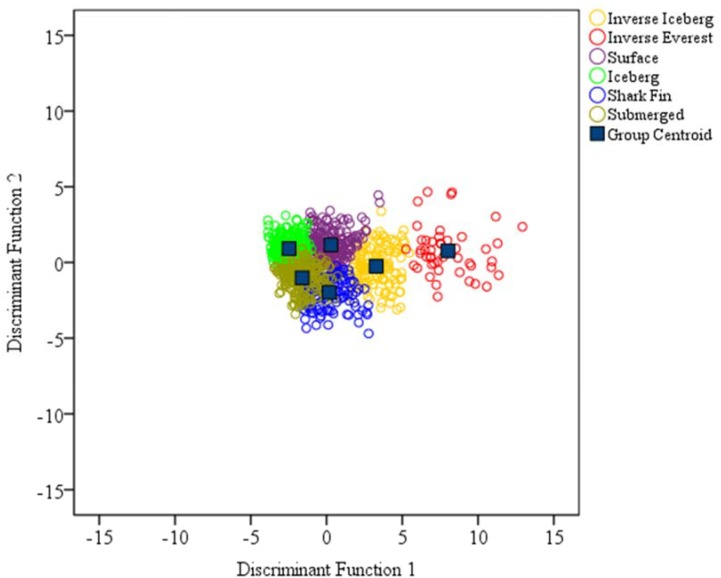
Distribution of cases in Dataset 1 (*N* = 929) for Functions 1 and 2.

As in Parsons-Smith et al. (2017), the five discriminant functions accounted for 100% of the variance, and each function predicted an outcome at a significant level (**Table 4**). **Figure 2** includes scatterplots of how the first two functions, which accounted for 95.7% of the variance, discriminated between clusters in Dataset 1. The scatterplots show the iceberg and submerged profiles to be the most tightly clustered around the cluster centroids, indicating that these profiles were the most clearly delineated clusters. By comparison the inverse Everest profile was relatively dispersed from the cluster centroid, indicating that it was the least clearly delineated cluster. This is also reflected in the high standard deviations associated with the inverse Everest profile (**Table 2**).

**Table 4:** Discriminant functions for Dataset 1 (*N* = 929) and Dataset 2 (*N* = 2,364).

Discriminant function	Eigenvalue	% of Variance	Cumulative %	Canonical correlation
**Dataset 1**				
1	6.935	80.8	80.8	0.935
2	1.276	14.9	95.7	0.749
3	0.310	3.6	99.3	0.487
4	0.056	0.7	99.9	0.230
5	0.005	0.1	100.0	0.069
**Dataset 2**				
1	5.678	71.3	71.3	0.922
2	1.693	21.3	92.5	0.793
3	0.498	6.2	98.8	0.576
4	0.094	1.2	99.9	0.293
5	0.004	0.1	100.0	0.067

A degree of overlap between the iceberg, submerged and shark fin profiles is apparent, which can be explained by the fact that low scores for tension, depression, anger, and confusion are common to all three clusters, which differ only according to the levels of vigor and fatigue reported. More specifically, the iceberg profile is characterized by high vigor and low fatigue, the submerged profile by low vigor and low fatigue, and the shark fin profile by low vigor and high fatigue.

To assess between-cluster differences in subscale scores among Dataset 1 (see **Table 2**) and by inference the independence of clusters derived from the ITAMS, a single-factor MANOVA was conducted, which showed a significant omnibus effect, Wilks’ Λ = 0.040, *F*(30, 929) = 151.65, *p* < 0.001, partial η^2^ = 0.475. Using a Bonferroni-adjusted alpha level of 0.008, significant univariate effects were confirmed for all mood dimensions: tension, *F*(5, 923) = 442.04, *p* < 0.001, partial η^2^ = 0.705; depression, *F*(5, 923) = 269.48, *p* < 0.001, partial η^2^ = 0.593; anger, *F*(5, 923) = 236.59, *p* < 0.001, partial η^2^ = 0.562; vigor, *F*(5, 923) = 173.42, *p* < 0.001, partial η^2^ = 0.484; fatigue, *F*(5, 923) = 255.71, *p* < 0.001, partial η^2^ = 0.581; and confusion, *F*(5, 923) = 252.97, *p* < 0.001, partial η^2^ = 0.578.

As shown in **Table 5**, the structure matrices of clusters recovered from the two datasets were very similar. Mood dimensions strongly associated with Function 1 included high tension, depression, confusion, anger, and fatigue, whereas Function 2 identified high vigor and low fatigue. Function 3 identified high fatigue and vigor, and Function 4 included high depression and low tension. Predictor variables associated with Function 5 varied between datasets. In Dataset 1, Function 5 included high confusion and low anger and tension, whereas in Dataset 2 it was associated with low confusion and depression, and high anger.

**Table 5:** Structure matrix for Dataset 1 (*N* = 929) and Dataset 2 (*N* = 2,364).

	Discriminant function				
Mood dimension	1	2	3	4	5
**Dataset 1**					
Tension	0.574	0.254	−0.085	−0.662^∗^	−0.309
Depression	0.453	0.057	−0.115	0.702^∗^	−0.295
Anger	0.425	0.129	−0.094	0.239	−0.455^∗^
Vigor	−0.132	0.739^∗^	0.631	0.048	−0.114
Fatigue	0.348	−0.542	0.741^∗^	−0.102	0.149
Confusion	0.427	0.264	−0.212	0.139	0.826^∗^
**Dataset 2**					
Tension	0.445	0.268	−0.063	−0.691^∗^	−0.126
Depression	0.560	0.149	−0.169	0.673^∗^	−0.303
Anger	0.494	0.234	−0.114	0.259	0.781^∗^
Vigor	−0.176	0.728^∗^	0.663	0.012	0.015
Fatigue	0.444	−0.545	0.706^∗^	−0.082	0.015
Confusion	0.546^∗^	0.250	−0.155	−0.255	−0.404

*^∗^Largest absolute correlation between each variable and any discriminant function.*

The classification table (see **Table 6**) showed that cluster membership was classified with a high degree of accuracy. The percentages of correct classifications were iceberg profile = 99.1%, inverse Everest profile = 95.7%, inverse iceberg profile = 97.7%, shark fin profile = 86.9%, submerged profile = 94.4%, and surface profile = 98.5%. Prior probabilities were 25.1, 5.1, 14.3, 13.1, 21.2, and 21.2%, respectively. The proportion of cases in each group was squared and summed (i.e., 0.251^2^ + 0.051^2^ + 0.143^2^ + 0.131^2^ + 0.212^2^ + 0.212^2^ = 0.193) to establish the proportional by chance accuracy rate. A total of 96.0% of the cases were correctly reclassified back into the original clusters; notably higher than the minimum classification accuracy rate of 44.3% (i.e., the proportional by chance accuracy rate +25%). This suggests that distribution overlap was small and the functions discriminated between clusters with a very high degree of accuracy.

**Table 6:** Cluster classifications for Dataset 1 (*N* = 929).

	Predicted group membership						
Cluster	1	2	3	4	5	6	*n*
Iceberg	231	0	0	0	1	1	233
Inverse Everest	0	45	2	0	0	0	47
Inverse iceberg	0	0	130	0	0	3	133
Shark fin	2	0	1	106	9	4	122
Submerged	9	0	0	0	186	2	197
Surface	1	0	1	0	1	194	197

*1, iceberg profile; 2, inverse Everest profile; 3, inverse iceberg profile; 4, shark fin profile; 5, submerged profile; 6, surface profile.*

Chi-squared tests were used to investigate differences in mood profiles according to gender and age group. Distributions were significantly different from expected values for both gender, χ^2^(5,923) = 27.26, *p* < 0.001, and age group, χ^2^(20,869) = 41.43, *p* = 0.003 (see **Table 7**). Adjusted residuals were then inspected to determine the source of the differences. As previously found in Dataset 2, males in Dataset 1 were significantly over-represented for the iceberg profile, while females were significantly over-represented for the more negative inverse Everest and shark fin profiles. There were no age differences relating to the inverse Everest, inverse iceberg, and submerged profiles. Consistent with Parsons-Smith et al. (2017), participants in Dataset 1 aged 18–24 were significantly under-represented for the iceberg profile, and significantly over-represented for the shark fin and surface profiles. Those in the 36–45 and 56–65 age groups were significantly over-represented for the iceberg profile, consistent with Dataset 2. The present findings related to gender and age group should be treated with caution due to violations of the underlying assumption of minimum cell counts for some categories of participants.

**Table 7:** Distribution of mood profile clusters by gender and age group for Dataset 1 (*N* = 929) and Dataset 2 (*N* = 2,364).

	Cluster source					
Gender	1	2	3	4	5	6
Male^1^	96^†+^	8^∗−^	31	25^∗−^	53	62
Female^1^	136^†−^	39^∗+^	101	97^∗+^	143	132
Male^2^	406^†+^	33	107^∗−^	196	288^∗−^	189
Female^2^	289^†−^	31	137^∗+^	213	315^∗+^	160
**Age group (year)**						
18–24^1^	113^†−^	29	85	84^∗+^	117	128^∗+^
25–35^1^	72^∗+^	14	31	28	49	46
36–45^1^	16^∗+^	2	4	2	12	4
46–55^1^	11^∗+^	0	3	1	7	2
56–65^1^	5^∗+^	0	0	0	4	0
18–24^2^	358^†−^	29^∗−^	151	274^§^^+^	374	230^∗+^
25–35^2^	110	22^†+^	33	54	89	48
36–45^2^	138^†+^	7	35	55	79	39^∗−^
46–55^2^	46	3	19	15^∗−^	35	20
56–65^2^	38^§^^+^	1	5	10	24	9
>65^2^	5	2^§^^+^	1	1	2	3

*1, iceberg profile; 2, inverse Everest profile; 3, inverse iceberg profile; 4, shark fin profile; 5, submerged profile; 6, surface profile. ^1^Dataset 1 (gender: N = 923; age group: N = 869), ^2^Dataset 2 (N = 2,364). ^+^over-represented, ^−^under-represented. ^∗^p < 0.05, ^§^ p < 0.01, ^†^p < 0.001.*

## Discussion

The primary aim of the present study was to determine whether the mood profile clusters identified by Parsons-Smith et al. (2017) among English-speaking samples would be reproduced in a different cultural and linguistic context using the ITAMS (Quartiroli et al., 2017). To facilitate comparison across the two cultural settings, findings based on the ITAMS data (Dataset 1) have been presented alongside findings from an archival BRUMS dataset interrogated by Parsons-Smith et al. (2017; Dataset 2). We can see from **Figure 1** that the six mood profile clusters identified in Dataset 1 traced similar patterns to the mood profile clusters in Dataset 2. The visual similarities were reinforced by the close match in the percentages of respondents in each cluster across the two datasets. A DFA yielded five functions accounting for 100% of the variance with the first two functions accounting for over 95.7% of the variance in Dataset 1 and 92.5% of the variance in Dataset 2 (see **Table 4**). The structure matrix of clusters recovered from each dataset was also very similar (**Table 5**).

Additional evidence for the cross-cultural replicability of these profile clusters can be found in their relationship with external variables, notably gender and age. These relationships were explored through chi-squared tests of independence between cluster membership and gender and age, respectively (**Table 7**). The distributions were not always identical across datasets but there were notable similarities. Males were over-represented and females were under-represented for the iceberg profile in both datasets. In relation to age, younger adults were under-represented and older adults were over-represented in the iceberg profile in both datasets. Additionally, younger adults were over-represented in the shark fin and surface profiles in both datasets.

The final comparison between the two datasets involved the mean scores on the six mood dimensions across the six clusters. Establishing the equivalence of profile clusters across the samples does not require the means for the two datasets to be equal but large differences would tend to create dissimilarities in the profiles. With such large samples, even small differences between means were significant so we searched instead for differences that represented large effects as assessed by Cohen’s *d* (Cohen, 1992). The only large effect (*d = 0.90*) involved the level of depression for the inverse Everest profile where the mean was much lower in Dataset 1 than Dataset 2.

On the basis of these findings we are satisfied that the six mood profile clusters are the same across the two datasets. Considering the major findings common to both datasets, foremost among these was the tendency for females and younger adults to be under-represented in the iceberg profile. There is no obvious explanation for this finding, especially considering that gender differences have not been a common feature of the BRUMS, as evidenced by the fact that the same set of norms is used for both males and females (Terry and Lane, 2010). It is possible that while gender differences on the individual mood dimensions of the various profiles are weak or non-existent, the profiles as a whole may capture emergent properties that show gender and age effects. This possibility should be explored in greater depth in future investigations.

### Limitations and Future Research

One limitation of the current study was that even with a large sample of 929 participants in Dataset 1, when distributed across six mood profiles and the various age groups, there were some empty cells and other cells with very small numbers (see **Table 7**). On the basis of findings from Parsons-Smith et al. (2017) and the current study, the less frequently reported profiles, such as the inverse Everest, require very large samples if they are to yield sufficient numbers to permit detailed analysis. With this in mind, and looking ahead to future research challenges in this field, two outstanding questions generated from the Parsons-Smith et al. (2017) study and the current study are whether the novel mood profiles are related to behavioral outcomes such as performance and risk of psychopathology, and whether populations are distributed in predictable ways across the profiles.

Addressing the first of these questions represents a clear direction for future investigations. A recent study conducted in an Italian context by Murgia et al. (2016), who investigated the mood states of 72 elite cyclists during a multistage race known as the Girobio, offers insights into how various profiles relate to athletic performance. Their results showed that both high and low performing cyclists reported iceberg profiles prior to the first race stage, indicating appropriate pre-competition mood (Beedie et al., 2000). Prior to the final race stage, at which point fatigue and other negative mood dimensions tended to become elevated, low performers typically reported inverse iceberg profiles whereas high performers tended to report surface profiles, suggesting that performance maintenance was associated with an ability to restrict the extent of mood decrements.

The finding that profile membership is related to gender and age group is encouragement to explore such relationships in greater depth, perhaps using archival datasets. The BRUMS and its other-language versions have been used extensively to explore the relationship between mood and performance. Researchers could interrogate existing data sets to identify the six clusters and investigate their relationships with performance. Regarding the distribution of populations across profiles, a feature of particular interest in the three samples used by Parsons-Smith et al. (2017) and the sample used in the current study was the similarity in the percentages of participants falling into each of the six mood profile clusters. Even the largest discrepancy between datasets, which occurred for the surface profile, represented only a six-percent difference in the incidence of the profile (**Figure 1**).

There is a need to continue to explore gender and age group variations in the incidence of specific mood profiles and also variations according to type of sport and other situational variables, using much larger datasets, possibly generated by different groups of researchers pooling their data. Given that the mean depression score for the inverse Everest profile (the most negative of the six profiles) was about one standard deviation lower in Dataset 1 than Dataset 2 (see **Figure 1**), another worthwhile line of enquiry would be to investigate whether this variation is a function of linguistic differences (Italian vs. English speakers) or the effects of regular physical activity (athletes/exercisers vs. general population).

## Conclusion

The accumulated visual and statistical evidence presented in the various tables and figures points to the replicability of the six mood profile clusters identified by Parsons-Smith et al. (2017), thus satisfying the primary aim of this study. The mood profile clusters identified in English-speaking samples were also identified in an Italian sample, supporting their cross-cultural generalizability and encouraging further exploration of mood profile clusters.

## Author Contributions

All authors listed have made a substantial, direct, and intellectual contribution to the work and approved it for publication.

## Conflict of Interest Statement

The authors declare that the research was conducted in the absence of any commercial or financial relationships that could be construed as a potential conflict of interest.

## Footnotes

1 www.moodprofiling.com
